# Tau Hypophosphorylation at Ser416 as the Early Molecular Imprint of Maternal Immune Activation: Insights from Female Mice Offspring

**DOI:** 10.3390/ijms262110778

**Published:** 2025-11-05

**Authors:** Ewelina Bielska, Marta Matuszewska, Piotr Wójcik, Anna Wilkaniec, Magdalena Cieślik, Magdalena Gąssowska-Dobrowolska, Dorota Sulejczak, Grzegorz A. Czapski, Agata Adamczyk

**Affiliations:** 1 Department of Cellular Signalling, Mossakowski Medical Research Institute, Polish Academy of Sciences, ul. Pawińskiego 5, 02-106 Warsaw, Poland; ebielska@imdik.pan.pl (E.B.); mmatuszewska@imdik.pan.pl (M.M.); pwojcik@imdik.pan.pl (P.W.); awilkaniec@imdik.pan.pl (A.W.); mcieslik@imdik.pan.pl (M.C.); mgassowska@imdik.pan.pl (M.G.-D.); aadamczyk@imdik.pan.pl (A.A.); 2 Department of Experimental Pharmacology, Mossakowski Medical Research Institute, Polish Academy of Sciences, ul. Pawińskiego 5, 02-106 Warsaw, Poland; dsulejczak@imdik.pan.pl

**Keywords:** Tau, neurodevelopmental disorders, cytokines, maternal immune activation, MIA, poly(I:C)

## Abstract

Maternal immune activation (MIA) is a recognized environmental risk factor for altered brain development, yet its early molecular consequences remain unclear. In this study, we examined total Tau, site-specific Tau phosphorylation, and selected synaptic proteins in one-month-old female mouse offspring exposed prenatally to MIA evoked by poly(I:C), a synthetic mimetic of viral dsRNA. Our analyses revealed a consistent reduction in Tau phosphorylation at Ser416 across multiple brain regions, including the cortex, hippocampus, and cerebellum, without changes in total Tau levels or other phosphorylation sites. Among synaptic markers, only Shank3 levels were decreased, and this effect was confined to the cerebellum. No additional robust alterations were detected at this stage of development. These findings suggest that Tau hypophosphorylation at Ser416 may represent an early and widespread molecular footprint of MIA, whereas cerebellar Shank3 downregulation points to a region-specific vulnerability of synaptic pathways. While the study is limited to female offspring and a single postnatal time point, the data provide new insights into subtle molecular signatures that could precede or accompany later functional outcomes. Our results highlight Tau phosphorylation and Shank3 expression as potential molecular markers of prenatal immune stress, warranting further longitudinal and sex-comparative studies to clarify their relevance for neurodevelopmental trajectories.

## 1. Introduction

Tau, a microtubule-associated protein (MAP), is canonically described as an axonal stabilizer of microtubules, regulating their dynamics and axonal transport. However, growing evidence indicates that Tau is also present in dendritic spines and presynaptic terminals, where it contributes to synaptic maturation and plasticity by modulating vesicle trafficking, receptor localization, and cytoskeletal dynamics [1,2]. Synaptic Tau exists as monomers, oligomers, or higher-order assemblies, whose localization and function are tightly regulated by post-translational modifications, particularly phosphorylation. Physiological phosphorylation supports plasticity and spine remodeling, whereas pathological phosphorylation promotes mislocalization, oligomerization, and synaptic dysfunction [2,3].

Tau’s functions extend beyond microtubules, as it interacts with a variety of synaptic proteins. At the presynaptic site, Tau can bind to vesicle membranes and proteins such as synaptophysin, synaptotagmin-1, and SNARE complex components, including syntaxin-1 and synaptobrevin [4]. These interactions may influence clathrin-mediated endocytosis and activity-dependent vesicle recycling, ultimately shaping release probability and neurotransmitter output [5,6,7]. Indeed, pathogenic Tau species reduce vesicle mobility and impair presynaptic function [7]. At the postsynaptic site, Tau engages multivalently with PSD-95, GKAP, Shank, and Homer, thereby altering the dynamics of PSD-like condensates and potentially stabilizing aberrant scaffold assemblies [8]. Genetic studies support a link between Tau and Shank3, showing that Shank3 deficiency synergizes with Tau pathology to exacerbate synaptic and cognitive deficits [9]. Since Shank1–3 and PSD-95 are central scaffolds at excitatory synapses, Tau-mediated disruption of these complexes may compromise receptor trafficking and plasticity, with particular relevance to neurodevelopmental disorders [10].

Together, these findings highlight Tau as a critical regulator of synaptic integrity, capable of coordinating spine dynamics, vesicle cycling, and receptor localization. Early disturbances in Tau function—including site-specific phosphorylation changes—may thus provide a mechanistic link between synaptic pathology and neurodevelopmental vulnerability.

Maternal immune activation (MIA) has been identified as a significant environmental risk factor for disorders such as autism spectrum disorder (ASD) and schizophrenia [11,12,13]. MIA is typically modeled in rodents using polyinosinic: polycytidylic acid (poly(I:C)) or lipopolysaccharide (LPS), which mimic viral and bacterial infections, respectively [14,15]. These agents act via pattern recognition receptors, such as TLR3 (poly(I:C)) or TLR4 (LPS), leading to activation of transcription factors (e.g., IRF3, AP-1, NF-κB) and increased cytokine production [16,17,18,19]. Cytokines such as IL-6, TNF-α, and IFN-γ are crucial for normal brain development [20,21,22,23,24]. However, their dysregulation during pregnancy can alter neuronal differentiation, microglial activation, and circuit formation [25,26,27]. Elevated maternal and fetal cytokine levels have been associated with increased risk of ASD and psychosis [28,29,30]. Although both MIA and Tau dysregulation have been independently implicated in neurodevelopmental disorders, our previous studies have explored their possible intersection [31].

As summarized above, Tau is not only a microtubule-associated protein implicated in neurodegeneration, but also a critical regulator of neuronal differentiation, axonal growth, and synaptic maturation during early brain development [32]. The dynamic regulation of Tau phosphorylation is essential for cytoskeletal remodeling and synaptic plasticity, indicating that even subtle disturbances in Tau function during development may exert long-lasting effects on neural circuit formation.

At the same time, MIA disrupts cytokine signaling and microglial activity during sensitive developmental periods, resulting in altered synaptic organization and behavioral phenotypes reminiscent of autism spectrum disorder [33,34]. Considering that inflammatory mediators such as IL-6, IL-1β, and TNF-α can directly influence Tau phosphorylation and microtubule stability [35], it is plausible that MIA-induced immune dysregulation leaves a persistent “molecular imprint” on Tau-related signaling pathways. Thus, the convergence between inflammatory and cytoskeletal mechanisms may represent an important link connecting maternal inflammation with synaptic dysfunction and neurodevelopmental vulnerability in offspring.

Although most studies on MIA have been conducted in male offspring, emerging evidence suggests that females may exhibit distinct and often subtler molecular and behavioral responses [36,37,38]. In the present study, we focused on female offspring to explore potential early molecular signatures associated with Tau regulation during neurodevelopment. This approach aims to provide an initial molecular framework for understanding early Tau-related alterations in females, a population that has been relatively underrepresented in previous MIA research, and to open new avenues for future investigations directly comparing both sexes.

Building on this rationale, we specifically sought to characterize early molecular changes in Tau and synaptic proteins in female offspring, providing a focused perspective on neurodevelopmental consequences of maternal immune activation.

Here, we investigate whether MIA in mice induces early alterations in Tau protein, its phosphorylation at distinct sites, and synaptic protein expression in one-month-old female offspring. We also compare brain and peripheral cytokine environments to examine whether central and systemic immune responses are aligned. To our knowledge, this is the first study to address Tau regulation in the context of MIA in female offspring, with particular attention to alterations in Tau phosphorylation as a potential early molecular signature.

## 2. Results

**Figure 1:**
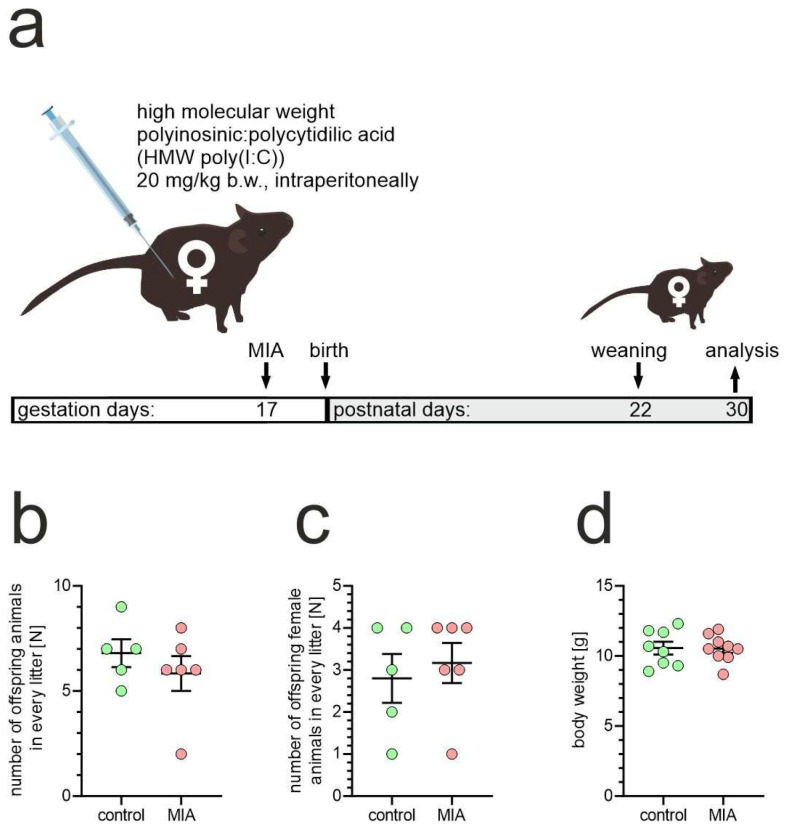
Characteristics of poly(I:C)-evoked MIA. (**a**) Experimental design. (**b**,**c**) The effect of poly(I:C)-evoked MIA on GD17 on pregnancy outcome in mice; n = 5 and 6 in control and MIA groups, respectively; every data point represents a separate litter. (**d**) The effect of poly(I:C)-evoked MIA on GD17 on the body weight of offspring animals; n = 8 in control group (distinct animals from 4 litters) and 9 in MIA group (distinct animals from 4 litters); every data point represents a separate animal. Means ± SEM were presented.

In our study, we utilized a mouse model of maternal immune activation (MIA) at late gestation to analyze molecular alterations in young offspring. High molecular weight poly(I:C), a mimetic of double-stranded RNA, was intraperitoneally injected into pregnant dams on gestation day 17 (GD17), then one-month-old female offspring animals were analyzed (Figure 1a) [39]. To evaluate if MIA could affect pregnancy outcome, we assessed the number of offspring from vehicle- or poly(I:C)-treated dams. As shown in Figure 1b,c, MIA did not affect the number of births. Also, the body weight of offspring was not affected by exposure to MIA (Figure 1d). We did not observe any obvious effect of MIA on the offspring’s state.

**Figure 2:**
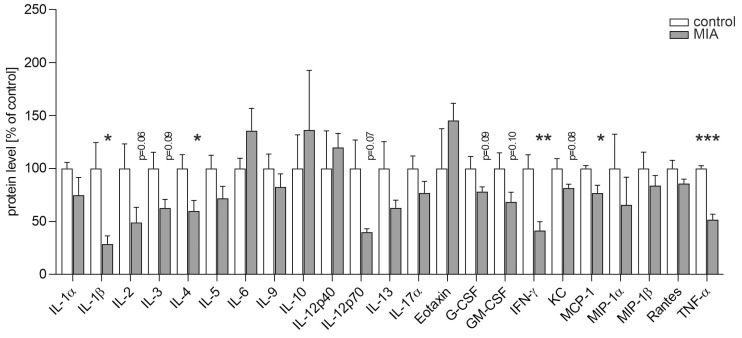
The effect of poly(I:C)-evoked MIA on the serum level of cytokines in one-month-old offspring. Poly(I:C) (20 mg/kg b.w.) was injected intraperitoneally at gestation day 17 to female mice. The blood was collected from one-month-old offspring female mice, and cytokine’ levels in blood serum were determined using a multiplex assay. Means ± SEM were presented. n = 4 in control group (distinct animals from 3 litters) and 5 in MIA group (distinct animals from 5 litters), except for IL-6 (n = 3 in control), IL-12p70 (n = 4 in MIA), TNF-α (n = 3 in control and 4 in MIA). Statistical analysis was performed using Student *t*-test. *, **, *** *p* < 0.05, *p* < 0.01, *p* < 0.001, respectively, compared to corresponding control.

Because MIA has been shown to induce long-term systemic reprogramming of immune function in both rodents and non-human primates [40,41,42,43], we first performed a multiplex analysis of 23 cytokines in blood serum to assess potential alterations in peripheral immune signaling. The results demonstrated that poly(I:C)-induced MIA elicited significant changes in the cytokine profile of one-month-old offspring. Although most cytokines showed a general tendency to decrease, statistically significant differences were observed in IL-1β, IL-4, IFN-γ, MCP-1, and TNF-α (Figure 2).

**Figure 3:**
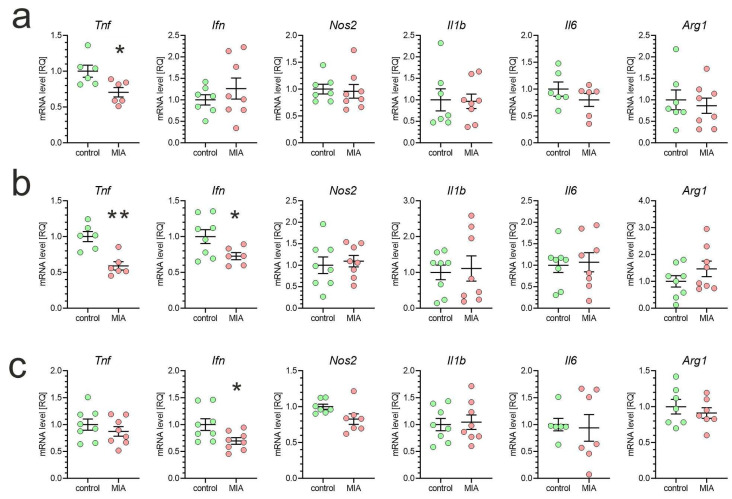
The effect of poly(I:C)-evoked MIA on gene expression of selected cytokines in the brain of one-month-old offspring. Poly(I:C) (20 mg/kg b.w.) was injected intraperitoneally at gestation day 17 into female mice. One-month-old female offspring mice were decapitated, and the brain tissue was collected. The levels of mRNA in the brain cortex (**a**), hippocampus (**b**), and cerebellum (**c**) were measured by using qPCR and calculated by the ΔΔCt method. Means ± SEM were presented. Group size (number of distinct animals/number of litters): cortex: control—6–7 animals/4 litters, MIA—6–8 animals/4 litters; hippocampus: control—6–8 animals/4 litters, MIA—6–8 animals/4 litters; cerebellum: control—6–8 animals/4 litters, MIA—7–8 animals/4 litters. Statistical analysis was performed using the Student’s *t*-test. *, ** *p* < 0.05, *p* < 0.01, respectively, compared to the corresponding control.

To analyze how MIA affects inflammatory signaling in the brain of offspring animals, we analyzed the mRNA levels of selected cytokines and inflammation-related genes in the brain cortex, hippocampus, and cerebellum. As shown in Figure 3, the expression of the *Tnf* gene was reduced in both the brain cortex and hippocampus, and the *Ifn* gene in the hippocampus and cerebellum. However, other tested inflammation-related genes (*Nos2*, *Il1b*, *Il6*) were not changed in MIA offspring. Additionally, the brain expression of the *Arg1* gene, which is considered a marker of protective, anti-inflammatory microglia, was unaffected by MIA.

**Figure 4:**
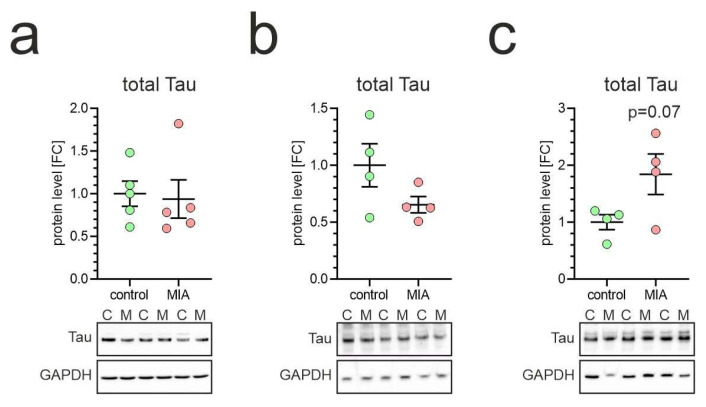
Effect of poly(I:C)-induced MIA on the protein level of Tau in the brains of one-month-old offspring. Poly(I:C) (20 mg/kg b.w.) was injected intraperitoneally into pregnant mice at gestation day 17. One-month-old female offspring were decapitated, and brain tissue was collected. Tau in cortex (**a**), hippocampus (**b**), and cerebellum (**c**) was analyzed by Western blot. Data are shown normalized to GAPDH. Means ± SEM were presented. Group size (number of distinct animals/number of litters): cortex: control—5 animals/3 litters, MIA—5 animals/2 litters; hippocampus: control—4 animals/3 litters, MIA—4 animals/2 litters; cerebellum: control—4 animals/3 litters, MIA—4 animals/4 litters. Statistical analysis was performed using Student’s *t*-test. Representative blots are shown (lower panel). C—control, M—MIA.

Recent studies show that Tau protein is expressed early during neuronal maturation in human fetal cortex and iPSC-derived cortical organoids, correlating with morphological development and increasing connectivity [44]. Moreover, genetic reduction of Tau in mouse brain impairs neuronal migration, dendritic growth, and synaptic bouton formation, implicating Tau as a key regulator of early brain architectural formation [45]. Our analysis revealed that Tau level was not affected in the tested brain structures of MIA-exposed animals (Figure 4). Likewise, we did not notice any significant change in the stoichiometry of Tau isoforms expressed in the tested brain structures in MIA animals, as compared to control animals.

**Figure 5:**
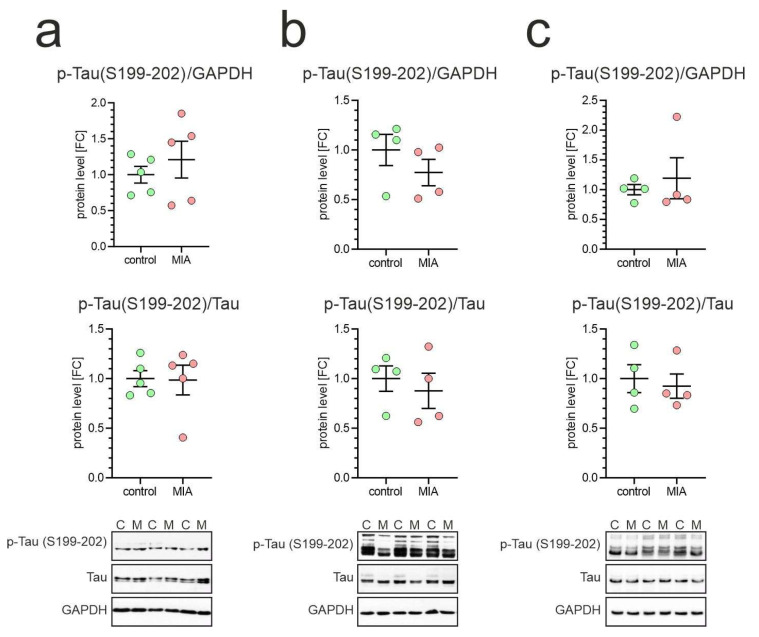
Effect of poly(I:C)-induced MIA on Tau phosphorylation at Ser199-202 in brains of one-month-old offspring. Poly(I:C) (20 mg/kg b.w.) was injected intraperitoneally into pregnant mice at gestation day 17. One-month-old female offspring were decapitated, and brain tissue was collected. Phospho-Tau (Ser199-202) levels in cortex (**a**), hippocampus (**b**), and cerebellum (**c**) were analyzed by Western blot. Data are shown normalized to GAPDH (**upper panel**) and to total Tau (**middle panel**). Means ± SEM are presented. Group size (number of distinct animals/number of litters): cortex: control—5 animals/4 litters, MIA—5 animals/4 litters; hippocampus: control—4 animals/4 litters, MIA—4 animals/4 litters; cerebellum: control—4 animals/4 litters, MIA—4 animals/4 litters. Statistical analysis was performed using Student’s *t*-test. Representative blots are shown (**lower panel**). C—control, M—MIA.

**Figure 6:**
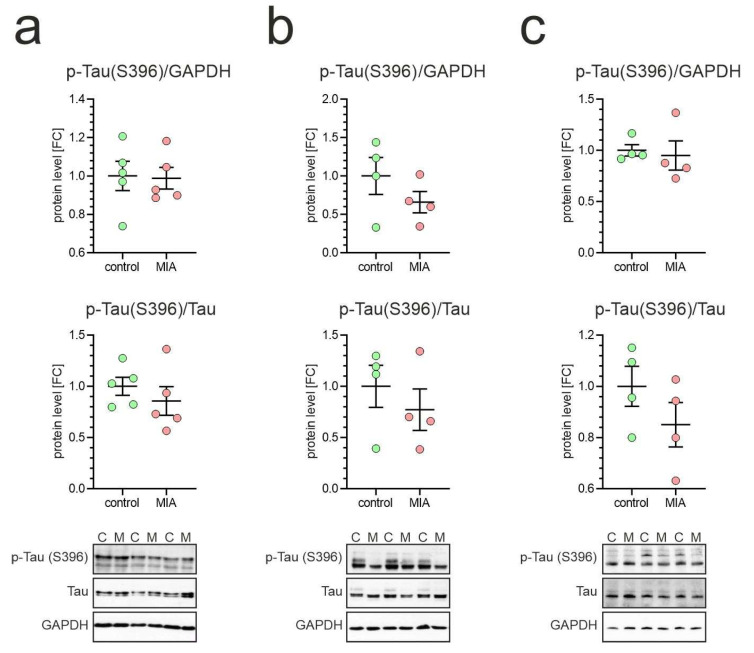
Effect of poly(I:C)-induced MIA on Tau phosphorylation at Ser396 in brains of one-month-old offspring. Poly(I:C) (20 mg/kg b.w.) was injected intraperitoneally into pregnant mice at gestation day 17. One-month-old female offspring were decapitated, and brain tissue was collected. Phospho-Tau (Ser396) levels in cortex (**a**), hippocampus (**b**), and cerebellum (**c**) were analyzed by Western blot. Data are shown normalized to GAPDH (**upper panel**) and to total Tau (**middle panel**). Means ± SEM are presented. Group size (number of distinct animals/number of litters): cortex: control—5 animals/2 litters, MIA—5 animals/3 litters; hippocampus: control—4 animals/2 litters, MIA—4 animals/3 litters; cerebellum: control—4 animals/4 litters, MIA—4 animals/3 litters. Statistical analysis was performed using Student’s *t*-test. Representative blots are shown (**lower panel**). C—control, M—MIA.

**Figure 7:**
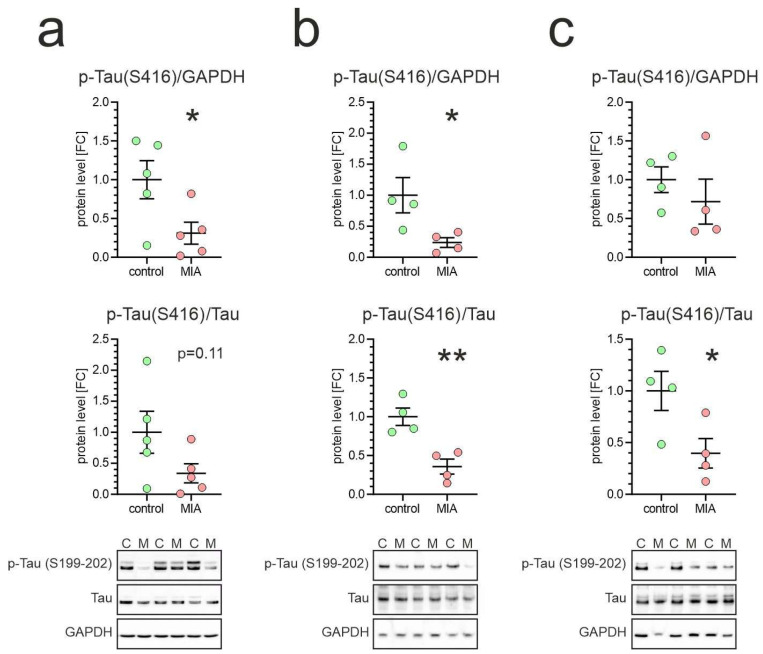
Effect of poly(I:C)-induced MIA on Tau phosphorylation at Ser416 in brains of one-month-old offspring. Poly(I:C) (20 mg/kg b.w.) was injected intraperitoneally into pregnant mice at gestation day 17. One-month-old female offspring were decapitated, and brain tissue was collected. Phospho-Tau (Ser416) levels in cortex (**a**), hippocampus (**b**), and cerebellum (**c**) were analyzed by Western blot. Data are shown normalized to GAPDH (**upper panel**) and to total Tau (**middle panel**). Means ± SEM are presented. Group size (number of distinct animals/number of litters): cortex: control—5 animals/3 litters, MIA—5 animals/2 litters; hippocampus: control—4 animals/3 litters, MIA—4 animals/2 litters; cerebellum: control—4 animals/3 litters, MIA—4 animals/4 litters. Statistical analysis was performed using Student’s *t*-test. *, ** *p* < 0.05, *p* < 0.01, respectively, compared to the corresponding control. Representative blots are shown (**lower panel**). C—control, M—MIA.

The analysis of Tau phosphorylation, as shown in Figure 5, Figure 6 and Figure 7, was performed using two complementary normalization strategies. Phospho-Tau immunoreactivity was first normalized to GAPDH immunoreactivity, providing a measure of the absolute phosphorylation level at the respective site. In parallel, phospho-Tau immunoreactivity was normalized to total Tau immunoreactivity, thereby indicating relative alterations in the proportion of Tau phosphorylated at the specific epitope. We performed an analysis of three important phosphorylation sites: Ser199-202, located in the proline-rich domain, and Ser396 and Ser416, located in the C-terminal domain. Phosphorylation of Tau at Ser199-202 controls microtubule-binding capacity; therefore, its dysregulation could promote cytoskeletal remodeling. Importantly, during early development, Tau is relatively highly phosphorylated at Ser199-202, and this changes over time [46,47,48]. Our analysis demonstrated that phosphorylation of Tau protein at Ser199-202 was not affected in MIA animals, as compared to control animals (Figure 5).

The phosphorylation of Tau at Ser396 (and PHF-1 epitope Ser396/404) is detectable in fetal and early postnatal brain, shows relatively moderate levels and remains largely stable from late prenatal stages into adulthood [48]. Alteration of the phosphorylation of Ser396 could affect Tau’s affinity for microtubules, potentially impairing their stabilization. Our results demonstrated that phosphorylation of Tau protein at Ser396 was not affected in MIA animals, as compared to control animals (Figure 6).

The level of Tau phosphorylation at Ser416 is notably elevated in the young, developing brain, but drops rapidly to barely detectable levels in seven-week-old rats [49]. At this point, the brain structure is established, and the stabilized microtubule network, facilitated by dephosphorylated Tau, is essential for maintaining the integrity and function of mature neurons. Our data showed a significant alteration in the level of Tau phosphorylation at Ser416. As shown in Figure 7, in every tested brain structure, the immunoreactivity of phospho-Tau(Ser416) was significantly reduced or showed some strong tendency to decrease.

**Figure 8:**
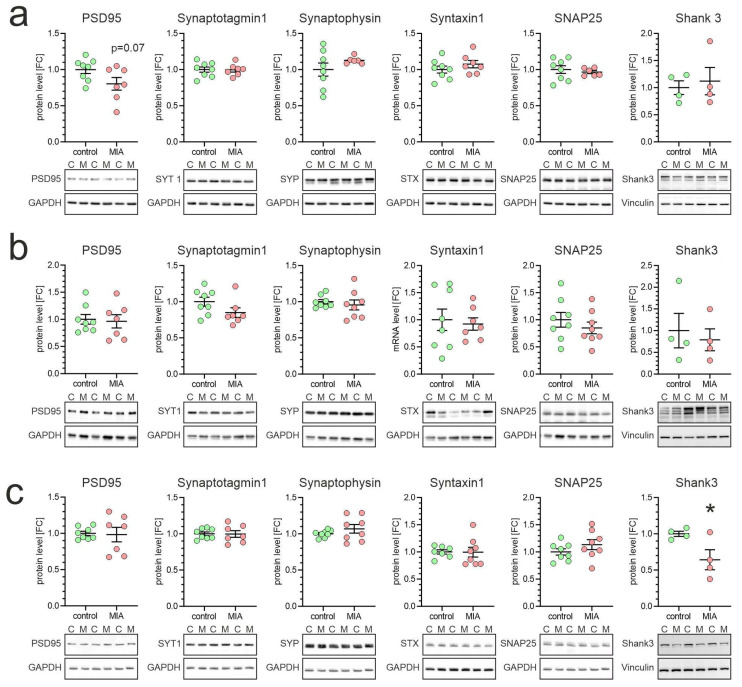
Effect of poly(I:C)-induced MIA on synaptic protein expression in brains of one-month-old offspring. Poly(I:C) (20 mg/kg b.w.) was injected intraperitoneally into pregnant mice at gestation day 17. One-month-old female offspring were decapitated, and brain tissue was collected. Synaptic protein levels in cortex (**a**), hippocampus (**b**), and cerebellum (**c**) were analyzed by Western blot. Densitometric values were normalized to GAPDH or to Vinculin (for Shank3). Data are presented as means ± SEM. For PSD95, Synaptotagmin1, Synaptophysin, Syntaxin1, and SNAP25, group size (number of distinct animals/number of litters): cortex: control—8 animals/4 litters, MIA—6–7 animals/4 litters; hippocampus: control—8 animals/4 litters, MIA—7–8 animals/4 litters; cerebellum: control—7–8 animals/4 litters, MIA—7–8 animals/4 litters. For Shank3, group size: cortex: control—4 animals/2 litters, MIA—4 animals/2 litters; hippocampus: control—4 animals/2 litters, MIA—4 animals/2 litters; cerebellum: control—4 animals/4 litters, MIA—4 animals/4 litters. Statistical analysis was performed using Student’s *t*-test. * *p* < 0.05 vs. control. Representative blots are shown. C—control, M—MIA.

Finally, we analyzed the impact of MIA on the level of synaptic proteins in offspring brains. As presented in Figure 8, MIA evoked a decrease in the Shank3 protein levels in the cerebellum.

## 3. Discussion

Prenatal factors increase the risk of developing neuropsychiatric and neurological disorders in offspring, most commonly autism and schizophrenia, but also neurodegenerative disorders and dementia [50,51,52]. In this study, we employed a late-gestation mouse model of MIA to examine how prenatal immune stress influences Tau protein levels, site-specific Tau phosphorylation, and synaptic protein expression in the brains of juvenile female offspring at one month of age.

We focused on female offspring to address a long-standing bias in experimental research, where the majority of studies—including those on MIA—have been conducted in males [53,54]. Given that females often display distinct and sometimes more subtle molecular and behavioral responses to prenatal insults, studying them provides a valuable and complementary perspective on the early effects of MIA [36,37,38,55,56]. Rather than aiming to compare the two sexes, our objective was to characterize early molecular events in females that may contribute to sex-divergent trajectories described in the literature [37,55,56]. This approach establishes a baseline for understanding MIA-induced alterations in the female brain and highlights the importance of including both sexes in future research to build a more complete picture of MIA-related pathophysiology. While this design precludes direct sex-based comparisons, it provides novel insight into molecular alterations in a relatively understudied population. We fully acknowledge that including male offspring in future studies will be essential to determine whether the observed changes are sex-specific or general features of maternal immune activation. The current discussion contrasts our findings with male-focused studies and frames our results as a complementary contribution to the broader literature.

We identified a significant, brain region–dependent reduction in the expression of key pro-inflammatory mediators, such as TNF-α and IFN-γ, in female offspring following MIA. This was accompanied by reduced levels of circulating pro-inflammatory cytokines (IL-1β, IFN-γ, MCP-1, TNF-α), as well as IL-4, a cytokine generally regarded as anti-inflammatory, which suppresses the production of classical pro-inflammatory mediators and promotes regulatory immune pathways. Cytokine levels typically fluctuate during development—for example, in the frontal and cingulate cortices, they rise at birth, decline during periods of synaptogenesis, and increase again in adulthood [19]. The present findings pinpoint a suppression of pro-inflammatory signaling during early postnatal life, a stage marked by intense synaptic plasticity. Whereas our previous work conducted in adult male MIA offspring demonstrated elevated TNF-α and IL-6 in the cerebral cortex, accompanied by increased serum concentrations of pro-inflammatory cytokines [27]. This agrees with reports showing that adult MIA offspring exhibit elevated macrophage-derived cytokines such as IL-12(p40) and CCL3, reflecting sustained M1-like inflammatory skewing [43]. Together, these observations suggest that MIA evokes dynamic, sex-, age-, and region-specific changes in cytokine expression during postnatal development.

Such a decrease may reflect a compensatory physiological adjustment that mitigates excessive inflammation during critical periods of neuronal differentiation and circuit formation, thereby supporting synaptic refinement and axonal growth. The concomitant reduction in the anti-inflammatory cytokine IL-4 suggests that this is not merely a suppression of inflammation, but rather a broader shift towards immune hyporesponsiveness. In this context, immune dysregulation may extend beyond heightened pro-inflammatory activity to include impaired regulatory mechanisms. Taken together, the data suggest that although cytokine levels in female offspring are suppressed early in postnatal development, MIA may still predispose them to an inflammatory rebound later in life, potentially contributing to neuroimmune and behavioral abnormalities in adulthood. Nonetheless, this possibility remains to be further investigated.

Importantly, MIA-evoked immune suppression in one-month-old offspring appears strongly dependent on the timing of the inflammatory insult. In general, pro-inflammatory challenges during early pregnancy elicit stronger and longer-lasting immune activation than those administered later [27,57]. Indeed, injection of poly(I:C) at gestational day 17 results in a potent but short-lived increase in maternal TNF-α levels, with concentrations already declining 6 h after injection compared to the 3 h peak, coinciding with a rise in the anti-inflammatory cytokine IL-10 [57]. This suggests the rapid activation of cytoprotective and immunosuppressive mechanisms, which may also shape the offspring’s immunity. The underlying mechanisms remain incompletely understood, but TLR3 signaling is a likely contributor. Poly(I:C) is a strong activator of TLR3, and in certain contexts (e.g., in mesenchymal stromal cells, MSCs), TLR3 activation induces immunosuppressive rather than pro-inflammatory responses [58].

MSCs, derived from both maternal and fetal sources, are thought to be central regulators of feto-maternal tolerance [59]. During early pregnancy, pro-inflammatory conditions dominate, which could be detrimental for the fetus, as its antigens might be recognized as foreign and trigger rejection [60]. MSCs counteract this by secreting immunosuppressive cytokines, thereby creating an anti-inflammatory milieu that can extend to the fetus. If excessively activated, MSC-driven suppression may persist into the neonatal period [61]. The reduction in pro-inflammatory cytokine levels observed in our study may reflect this mechanism. Furthermore, MSCs inhibit Th1 lymphocyte and M1 macrophage differentiation, thereby biasing immunity towards Th2 and M2 responses [59,62,63,64], which would prolong the anti-inflammatory state. As MSCs are potent producers of IL-10 [65,66], the increase in the IL-10:IFN-γ and IL-10:TNF-α ratios observed here likely reflects this shift. Interestingly, the IL-10:IL-6 ratio remained stable, consistent with the fact that both IL-10 and IL-6 are abundantly secreted by MSCs [66]. Together, these observations support the hypothesis that TLR3 activation in MSCs drives immunosuppressive activity after poly(I:C) administration and may constitute a key regulatory axis shaping early-life immunity.

The most striking outcome of our study is the selective reduction in Tau phosphorylation, positioning Tau as a primary molecular target of prenatal immune challenge. Although Tau dysfunction is canonically associated with neurodegenerative disease (e.g., Alzheimer’s disease), converging evidence indicates that Tau phosphorylation is also implicated in neurodevelopmental disorders. Abnormalities in Tau, including aberrant phosphorylation and altered expression, have been described in ASD [67,68]. A landmark study by Tai and co-workers [13] demonstrated that Tau reduction prevents autism-like behaviors in mouse models, establishing Tau not only as a cytoskeletal regulator but also as a potential therapeutic target in ASD.

Our previous work using male offspring supports this view: in the valproic acid (VPA) rat model of ASD, we observed increased cortical Tau phosphorylation at Ser396 and Ser416, accompanied by microtubule (MT) destabilization and autistic-like behaviors [69]. In the cerebellum of VPA-exposed males, Tau levels were reduced alongside decreased pSer416 but increased pSer396, indicating brain region–specific Tau dysregulation [70]. By contrast, in the MIA model induced by LPS, hippocampal Tau levels remained stable, with unchanged pSer416 but significant reductions in pSer199/202 and pSer396 phosphorylation [31]. In a transgenic tuberous sclerosis complex (TSC) mouse model, we also observed marked, structure-specific changes in Tau expression and phosphorylation linked to MT disruption and behavioral deficits in males [71]. Collectively, these findings underscore Tau’s dual role as a cytoskeletal regulator and a sensitive responder to inflammatory and developmental perturbations in males, whereas the present data extend this concept to females, revealing reduced Tau phosphorylation without accompanying increases in total Tau. To our knowledge, Tau alterations have not previously been investigated in female MIA offspring.

Tau function is tightly regulated by phosphorylation, particularly within its C-terminal tail region (residues 368–441). Phosphorylation at Ser396 modulates the Tau–MT binding affinity, while phosphorylation at Ser416 couples neuronal activity and proteostasis to MT engagement. Additionally, phosphorylation at Ser199/202 reshapes axonal transport and MT architecture through conformational control [72]. In the present study, total Tau levels in female offspring remained unchanged in the cerebral cortex and hippocampus, with only a trend toward increased expression in the cerebellum. Notably, however, we observed a selective reduction in Tau pSer416 in the cortex and hippocampus—regions where TNF-α expression was significantly decreased. In the cerebellum, pSer416 levels displayed a downward trend when normalized to GAPDH and a significant reduction when normalized to total Tau, clearly indicating altered phosphorylation dynamics rather than a simple reduction in pSer416 abundance.

An imbalance between kinase and phosphatase signaling may underlie these changes. Ca^2+^/calmodulin-dependent protein kinase II (CaMKII), the primary kinase targeting Serine 416, plays a crucial role in ASD-related behaviors [73]. Conversely, protein phosphatase type 2A (PP2A) is the predominant Tau phosphatase, and its dysfunction has been implicated in multiple neurodevelopmental disorders, including ASD [74]. A relative reduction in CaMKII activity or enhanced PP2A-mediated dephosphorylation, potentially downstream of altered cytokine environments (e.g., decreased TNF-α), could explain the diminished phosphorylation at Ser416 observed in this study. Functionally, reduced Ser416 phosphorylation may bias Tau toward more stable MT binding, thereby supporting axonal transport and cytoskeletal integrity. In very young female offspring, this shift may act as a compensatory mechanism that buffers against synaptic or axonal vulnerabilities otherwise observed in ASD models. This scenario aligns with growing evidence that female offspring display distinct molecular and behavioral trajectories after MIA, in some cases exhibiting resilience or adaptive responses. Recent findings further indicate that MIA leaves long-lasting cytoskeletal imprints in neural stem cells [75], suggesting that Tau regulation may represent one key downstream mechanism of such developmental reprogramming.

To determine whether Tau hypophosphorylation coincides with synaptic alterations, we assessed core postsynaptic proteins. Our analyses revealed a selective reduction in Shank3 protein in the cerebellum, without corresponding changes in the cortex or hippocampus, and with no alterations in other postsynaptic density proteins such as PSD95. SHANK proteins (Shank1–3) are central scaffolding molecules of the postsynaptic density (PSD), coordinating receptor complexes, cytoskeletal elements, and signaling proteins. Shank3, in particular, has been repeatedly linked to neurodevelopmental disorders, most prominently autism. Mutations or deletions in Shank3 cause Phelan–McDermid syndrome, characterized by global developmental delay, intellectual disability, and high ASD prevalence [76,77,78]. Rare disruptive Shank3 variants are also enriched in idiopathic ASD [79,80,81]. However, mapping studies show heterogeneous Shank3 expression across brain regions and patient cohorts, with isoform- and region-specific alterations [82].

Animal models with Shank3 deletions reliably recapitulate ASD-like phenotypes; however, Shank3 levels vary across different brain regions and developmental stages [76,79,83]. Our present results align with earlier work showing that MIA alters SHANK expression [31]; however, in this study, the reduction was specifically observed in the cerebellum of young female offspring. In contrast, adult male rats prenatally exposed to LPS-induced MIA exhibited broader synaptic disturbances, characterized by marked downregulation of Shank3 [31]. Thus, Shank3 appears to be a particularly sensitive molecular target of MIA, with its dysregulation emerging earlier than that of other synaptic proteins, but in a region- and sex-dependent manner. Reducing Shank3 exclusively in the cerebellum may destabilize local synaptic architecture and plasticity, contributing to ASD-related phenotypes later in life. Nevertheless, MIA effects are not uniform and may interact with genetic predispositions. In a dual-hit model combining Shank3Δ11 deficiency with poly(I:C)-induced MIA, [84] unexpectedly reported upregulation of several PSD proteins, including Shank3, suggesting that MIA exacerbates synaptic imbalance through diverse mechanisms, not solely by reducing Shank3 levels.

In summary, our findings identify Tau hypophosphorylation at Ser416 as the earliest and most consistent molecular imprint of maternal immune activation in female offspring. Rather than reflecting a state of sustained inflammation, this signature emerges within a transiently suppressed immune milieu and coincides with selective synaptic vulnerability, notably a reduction in Shank3 in the cerebellum. By highlighting Tau as a central integrator of prenatal immune stress, cytoskeletal regulation, and synaptic integrity, this work reframes early postnatal development as a critical window in which protective and pathological processes diverge, with important implications for understanding sex-specific resilience and risk in neurodevelopmental disorders.

Despite these advances, several limitations must be considered. The mechanistic basis of Tau hypophosphorylation remains unresolved. Although our results are consistent with altered kinase–phosphatase signaling, other factors, such as microglial phenotypes or intrinsic developmental trajectories, may also contribute. Moreover, our study focused exclusively on female offspring. While this provides novel insight into a relatively understudied population, it limits the ability to draw conclusions about potential sex differences. Future work, including male offspring, will be essential to determine whether the observed molecular alterations are sex-dependent or reflect general features of maternal immune activation. In addition, our analyses capture a single postnatal time point, limiting the ability to assess the dynamics of Tau phosphorylation and cytokine expression across development. Longitudinal studies will be needed to establish whether early hypophosphorylation is transiently protective or predisposes to later vulnerability. Finally, although we identified parallel changes in Tau and Shank3, the functional consequences for synaptic physiology and behavior remain to be clarified. Future experiments combining molecular, electrophysiological, and behavioral approaches will be necessary to define how these molecular alterations shape neurodevelopmental trajectories. Nevertheless, this study broadens current knowledge of MIA-related effects in female offspring and provides a foundation for future investigations. The subtle and limited alterations observed in the synaptic protein panel suggest that, at this early stage, molecular reconfiguration of Tau may precede more extensive disruptions of synaptic components. Given that Tau exerts both presynaptic and postsynaptic functions in development and plasticity, hypophosphorylation could represent an early mechanism modulating synaptic structure and dynamics before broader synaptic alterations become evident. This perspective aligns with previous reports highlighting the non-canonical yet functionally relevant presence of Tau in synaptic signaling, and it underscores the value of studying female offspring as a complementary approach to existing male-focused MIA research [3].

## 4. Materials and Methods

### 4.1. Materials

Reagents for reverse transcription (High-Capacity cDNA Reverse Transcription Kit with RNase Inhibitor) and quantitative PCR (Taqman Assays, and TaqMan Fast Advanced Master Mix) were obtained from Thermo Fisher Scientific, Inc. (Waltham, MA, USA). High molecular weight (average size of 1.5–8 kb) polyinosinic-polycytidylic acid (HMW poly(I:C)) was from InvivoGen (San Diego, CA, USA). TRI-reagent, DNase I, dithiothreitol (DTT), anhydrous dimethyl sulfoxide (DMSO) and all other reagents were obtained from Sigma-Aldrich (St. Louis, MO, USA). Mouse anti-PSD95 Ab, mouse anti-Synaptophysin Ab, mouse anti-Syntaxin1 Ab, mouse anti-Shank3 Ab, mouse anti-Tau Ab were from Santa Cruz Biotechnology. Rabbit anti-Vinculin Ab, rabbit anti-Synaptotagmin1 Ab, rabbit anti-Snap25 Ab, mouse anti-pTau(Ser396) Ab, and rabbit anti-pTau(Ser416) Ab were from Cell Signaling Technology (Danvers, MA, USA), and rabbit anti-pTau(Ser199-202) Ab were from Sigma-Aldrich (St. Louis, MO, USA). Secondary antibodies: anti-mouse IgG was from Thermo Fisher Scientific, Inc. (Waltham, MA, USA), and anti-rabbit IgG was from Sigma-Aldrich. HRP-conjugated GAPDH Ab was from Proteintech Europe Ltd. (Manchester, UK). Bio-Plex Pro Mouse Cytokine 23 assay kit was from Bio-Rad Laboratories (Hercules, CA, USA).

### 4.2. Animals

The experiments were carried out on C57BL/6J mice supplied by the Animal House of Mossakowski Medical Research Institute, Polish Academy of Sciences (Warsaw, Poland), which operates breeding of small rodents with the SPF standard. The animals were maintained under controlled temperature and humidity conditions on a 12-h light/dark cycle in open polycarbonate cages in an enriched environment (plastic shelters, wooden blocks, wood shavings, and cotton pads as nesting material). All of the experiments conducted on animals were approved by the Local Ethics Committee for Animal Experimentation in Warsaw (protocol code WAW2/052/2021; issued on 21 April 2021) and were carried out in accordance with the EU Directive 2010/63/EU for animal experiments. Every effort was made to minimize the number of animals used and reduce the amount of pain and distress. All experimental procedures were performed between 8:00 and 12:00.

### 4.3. Experimental Design

The mice pregnancies were achieved by housing a male and a female overnight. The next morning, female mice were separated. Pregnant female mice were identified and transferred to the experimental breeding facility for acclimatization. All treatments were performed in the experimental room. MIA was evoked by intraperitoneal (i.p.) administration of HMW poly(I:C) (20 mg/kg b.w.) at gestation day 17 (GD17) [85,86]. Females from the control group received i.p. administration of analogous volume of vehicle (sterile 0.9% NaCl). Allocation to experimental groups was random. All dams were allowed to give birth and nurture offspring under normal conditions. In whole experiment, 11 dams (5 in control group and 6 in MIA group) gave birth of 33 female offspring (14 in control group and 19 in MIA group). On postnatal day (PND) 22 to 23, female pups were separated and housed in groups of 3 or 4 in open polycarbonate cages in an enriched environment. At one month of age, all offspring female mice from the control and MIA groups were anesthetized by isoflurane inhalation and blood and brain tissues (decapitation) were collected. Immediately after formation of clot, blood samples were centrifuged at 1000× *g* for 5 min to separate the serum. All tissue and serum samples were snap frozen and stored at −80 °C until analysis. Samples for analysis were randomly selected. No a priori exclusion criteria were defined. Operators were not blinded to the experimental group. All offspring male mice were assigned to our aging-related project.

### 4.4. Analysis of Serum Cytokine Levels

The level of cytokines in the serum was determined using multiplex technology [31]. Before analysis, all samples were diluted four times with dedicated sample diluent. The level of 23 cytokines (IL-1α, L-1β, IL-2, IL-3, IL-4, IL-5, IL-6, IL-9, IL-10, IL-12 p40, IL-12 p70, IL-13, IL-17A, Eotaxin, G-CSF, GM-CSF, IFN-γ, KC, MCP-1, MIP-1α, MIP-1β, RANTES, TNF-α) was determined by using Bio-Plex Pro™ Mouse Cytokine 23-Plex Assay on the Luminex Bio-Plex 200 system (Bio-Rad Laboratories, Hercules, CA, USA) according to the manufacturer’s instructions. Data calculation was based on a calibration curve obtained with recombinant cytokines.

### 4.5. Western Immunoblotting

Analysis of immunoreactivity of proteins was performed as described previously [31]. Tissue samples were homogenized in RIPA buffer, and protein concentration was determined using the Pierce™ BCA Protein Assay Kit (Thermo Fisher Scientific, Inc., Waltham, MA, USA) according to the manufacturer’s instructions, with BSA as a standard. Samples were mixed with Laemmli buffer and denatured at 95 °C for 5 min. After SDS-PAGE, proteins were transferred in standard conditions to a nitrocellulose membrane and used for immunochemical analysis, followed by chemiluminescent detection (Bio-Rad Laboratories, Hercules, CA, USA). Densitometric analysis was performed using normalization to glyceraldehyde 3-phosphate dehydrogenase (GAPDH) level for small and medium-size proteins and using normalization to Vinculin for Shank3 which is a large protein (180–200 kDa). Size-marker based verification was performed with ImageLab 6.1 software (Bio-Rad Laboratories, Hercules, CA, USA).

### 4.6. Analysis of the mRNA Level

Analysis of mRNA level was performed as described previously [87]. Total RNA was isolated by using TRI reagent according to the manufacturer’s protocol. Digestion of possible DNA contamination was performed by using DNase I according to the manufacturer’s protocol (Sigma-Aldrich, St. Louis, MO, USA). The quality and quantity of RNA were measured by spectrophotometric analysis. Reverse transcription was performed using a High-Capacity cDNA Reverse Transcription Kit with RNase Inhibitor according to the manufacturer’s protocol (Thermo Fisher Scientific, Inc., Waltham, MA, USA). Quantitative PCR was per-formed on an ABI PRISM 7500 apparatus by using TaqMan Fast Advanced Master Mix and the commercially available TaqMan Gene Expression Assays: *Gusb*-Mm01197698_m1, *Tnf*-Mm00443258_m1, *Il1b*-Mm00434228_m1, *Nos2*-Mm00440502_m1, *Arg1*-Mm00475988_m1, *Ifn*-Mm01168134_m1, *Il6*-Mm00446190_m1. The relative levels of mRNA were calculated by using the ΔΔCt method using *Gusb* as a reference gene

### 4.7. Statistical Analysis

The group size was calculated with G*Power 3.1. software (https://www.psychologie.hhu.de/arbeitsgruppen/allgemeine-psychologie-und-arbeitspsychologie/gpower; accessed on 22 March 2021), which calculates the minimum required group size based on the size of the Cohen d effect. Standard assumptions adopted: test power = 0.95, significance level = 0.01, groups of equal size. To reduce the risk of litter effect, animals from several litters in each experimental group (random selection) were tested. The results were expressed as mean values ± SEM. The statistical analysis of data was performed by using GraphPad Prism version 8.3.0 (GraphPad Software, San Diego, CA, USA). The significant outliers were excluded. Distribution of data was analyzed using Shapiro–Wilk test. Data were analyzed using Student’s *t*-test. *p* values <0.05 were considered significant. The *n* refers to independent animal samples, except in Figure 1b,c, where each data point represents a litter from an independent dam.

## 5. Conclusions

Our study reveals a region-specific hypophosphorylation of Tau in one-month-old female offspring following maternal immune activation (MIA), uncovering a previously unrecognized, developmentally regulated role of Tau in the early postnatal brain. Rather than serving solely as a marker of pathology, Tau appears to function as a molecular integrator of prenatal immune experience, encoding an early biochemical imprint of neurodevelopmental risk.

The observed Tau hypophosphorylation, together with reduced pro-inflammatory cytokine levels and only subtle synaptic alterations, suggests that modulation of Tau phosphorylation represents a core molecular consequence of prenatal immune challenge in females. Although our analyses were limited to female offspring and an early developmental stage, these findings highlight a potentially sex-specific and time-sensitive mechanism through which maternal immune activation may influence the trajectory of brain maturation. Future longitudinal and comparative studies will be critical to determine whether these changes persist across development or manifest differently in males.

Collectively, our results position Tau as a dynamic molecular link between the maternal immune milieu and offspring neurodevelopment, providing new conceptual ground for understanding how early immune perturbations shape the molecular architecture of the developing brain and its long-term functional outcomes.

## Data Availability

The original contributions presented in this study are included in the article. Further inquiries can be directed to the corresponding author.

## Abbreviations

The following abbreviations are used in this manuscript:

ASD	Autism spectrum disorder
AD	Alzheimer’s disease
BSA	bovine serum albumin
HMW	High molecular weight
LPS	lipopolysaccharide
MIA	maternal immune activation
MT	microtubule
poly(I:C)	polyinosinic-polycytidylic acid
PND	postnatal day
PSD	postsynaptic density
TSC	tuberous sclerosis complex
VPA	valproic acid
